# A unified spectrum model for the anti-GQ1b antibody syndromes: from pathophysiology to a new diagnostic framework

**DOI:** 10.3389/fneur.2026.1763283

**Published:** 2026-04-21

**Authors:** Khalil El Abdi, Fazeela Bibi, Muhammad Ibrahim, Kanza Ahmed, Suraksha Nagdev, Zulekhan Bibi, Bilal Aslam, Vohra Maham Hassan, Muhammad Sarim Azad Khan, Asghar Shah, Muhammad Saad Sammi, Abdullah Khan, Hania Imran, Said Hamid Sadat

**Affiliations:** 1 Faculty of Medicine and Pharmacy of Rabat, Mohammed V University, Rabat, Morocco; 2 Jinnah Medical and Dental College, Karachi, Pakistan; 3 Bannu Medical College, Bannu, Pakistan; 4 United Medical and Dental College, Karachi, Pakistan; 5 Shaheed Mohtarma Benazir Bhutto Medical University, Larkana, Pakistan; 6 Changsha Medical University, Changsha, China; 7 University of Lahore, Lahore, Pakistan; 8 Combined Military Hospital, Lahore Medical College, Lahore, Pakistan; 9 Khyber Teaching Hospital, Peshawar, Pakistan; 10 Shenyang Medical College, Shenyang, China; 11 Wah Medical College, Wah Cantt, Pakistan; 12 Kabul University of Medical Sciences Abu Ali Ibn Sina, Kabul, Afghanistan

**Keywords:** antibody, anti-GQ1b, Bickerstaff, brainstem, encephalitis, Fisher, Guillain-Barré syndrome, Miller

## Abstract

**Background:**

Bickerstaff Brainstem Encephalitis (BBE) is a rare, post-infectious autoimmune disorder characterized by ophthalmoplegia, ataxia, and altered consciousness. Its significant clinical and serological overlap with Miller Fisher syndrome (MFS) and Guillain-Barré syndrome (GBS) creates diagnostic challenges, particularly in atypical or seronegative presentations. These conditions are collectively understood as components of the anti-GQ1b antibody syndrome, a spectrum of neuro-immune disorders.

**Aims:**

This review provides a clinically focused, integrative synthesis of the anti-GQ1b antibody spectrum, with an emphasis on BBE. We aim to clarify the underlying pathophysiology, delineate the range of clinical phenotypes, provide a structured framework for diagnosis that acknowledges the limitations of ancillary testing, and summarize current evidence for therapeutic strategies.

**Synthesis of evidence:**

Pathogenesis is primarily driven by anti-GQ1b IgG antibodies, generated following infection via molecular mimicry, which trigger complement-mediated damage to ganglioside-rich neural structures. The clinical presentation ranges from purely peripheral deficits (MFS) to severe central nervous system dysfunction (BBE), with frequent BBE-GBS overlap syndromes. Diagnosis hinges on clinical recognition, supported by serology, with neuroimaging serving a crucial role in excluding mimics. Approximately one-third of clinically defined BBE cases are seronegative, suggesting alternative mechanisms. Prompt immunotherapy with intravenous immunoglobulin (IVIg) or plasma exchange is the cornerstone of management, extrapolated from GBS trials, while evidence for corticosteroids remains limited.

**Conclusion:**

A structured, spectrum-based perspective remains essential for clinicians navigating the diagnostic complexities of the anti-GQ1b antibody syndromes. This updated synthesis is intended to enhance diagnostic accuracy, guide therapeutic reasoning across the full range of phenotypes, and highlight key unresolved questions to inform a future research agenda.

## Introduction

Bickerstaff Brainstem Encephalitis (BBE) is an uncommon post-infectious autoimmune disorder characterized by the clinical triad of a rapid decline into ophthalmoplegia, ataxia, and altered consciousness (1–3). The landmark discovery of IgG anti-GQ1b antibodies fundamentally linked BBE’s pathophysiology to Miller Fisher syndrome (MFS) and Guillain-Barré syndrome (GBS) (4), establishing these conditions as core components of the “anti-GQ1b antibody syndrome”—a spectrum of disorders rooted in molecular mimicry following an antecedent infection (5, 6). These syndromes are rare, though precise epidemiological data are limited. The annual incidence of BBE is estimated to be approximately 0.08 per 100,000, making it significantly less common than GBS (1–2 per 100,000) and MFS (approximately 0.1 per 100,000) (5, 6).

The concept of a continuous clinical and immunopathological spectrum unified by the anti-GQ1b antibody is well-established within the neuroimmunology community (7, 8). However, for the practicing clinician, navigating this spectrum remains a diagnostic and therapeutic challenge. The marked phenotypic heterogeneity, which ranges from isolated cranial nerve palsies to fulminant encephalopathy with quadriparesis, often creates significant nosological ambiguity (9). This challenge is amplified by the existence of seronegative presentations, which account for up to one-third of clinically defined BBE cases, and by the limitations of ancillary investigations, particularly in the acute phase of the illness (4, 10–12). It is these persistent clinical challenges that justify the need for an updated, integrative synthesis of the available evidence.

The purpose of this review is therefore not to propose a new paradigm, but to provide a clinically focused synthesis of the anti-GQ1b antibody spectrum, with a particular emphasis on BBE. We aim to offer a structured framework for clinicians that: (1) synthesizes the clinical, serological, and neuroimaging evidence supporting a unified spectrum model; (2) proposes a practical, integrated diagnostic approach that acknowledges key limitations and pitfalls; and (3) critically appraises current therapeutic strategies by linking them to the underlying mechanisms of complement-mediated damage.

## Literature search and synthesis strategy

This article provides a narrative review designed to synthesize the clinical and pathophysiological evidence for the anti-GQ1b antibody spectrum of disorders. The selection of literature was guided by a structured, expert-driven curation process, and the manuscript’s preparation was informed by the principles outlined in the Scale for the Assessment of Narrative Review Articles (SANRA) to ensure transparency and rigor.

### Information sources and search strategy

A comprehensive literature search was conducted using the PubMed/MEDLINE, Scopus, and Cochrane Library databases for articles published from 1951 through February 2026. The search utilized a combination of keywords and MeSH terms, including “Bickerstaff Brainstem Encephalitis,” “anti-GQ1b antibody syndrome,” “Miller Fisher syndrome,” “Guillain-Barré syndrome overlap,” and “rhombencephalitis.” To ensure a complete evidence base, the reference lists of key articles and prior systematic reviews were also manually screened to identify additional relevant literature.

### Eligibility criteria

Articles were selected for inclusion based on the following criteria:

- Inclusion criteria:

◦ Population and content: Studies involving human subjects that focused on the epidemiology, immunopathogenesis, clinical presentation, neuroimaging, diagnosis, or management of the anti-GQ1b antibody syndrome and its core clinical entities (Bickerstaff Brainstem Encephalitis, Miller Fisher Syndrome, and related overlap syndromes).
◦ Publication type: Prioritization was given to systematic reviews, meta-analyses, large prospective or retrospective case series, and seminal pathophysiological research. Case reports and smaller case series were included selectively to illustrate the full breadth of clinical phenotypes, document atypical or seronegative presentations, and trace the historical evolution of the syndrome’s understanding.
◦ Language: The article was published in English.

- Exclusion criteria:

◦ Articles published in a language other than English.
◦ Articles for which the full text could not be retrieved.
◦ Publication types such as editorials, letters to the editor, and conference abstracts, unless they contained unique primary data essential for the review’s scope.
◦ Studies focused on non-human subjects, except for foundational mechanistic studies cited to explain core pathophysiology.

### Study selection and data synthesis

**Figure 1:**
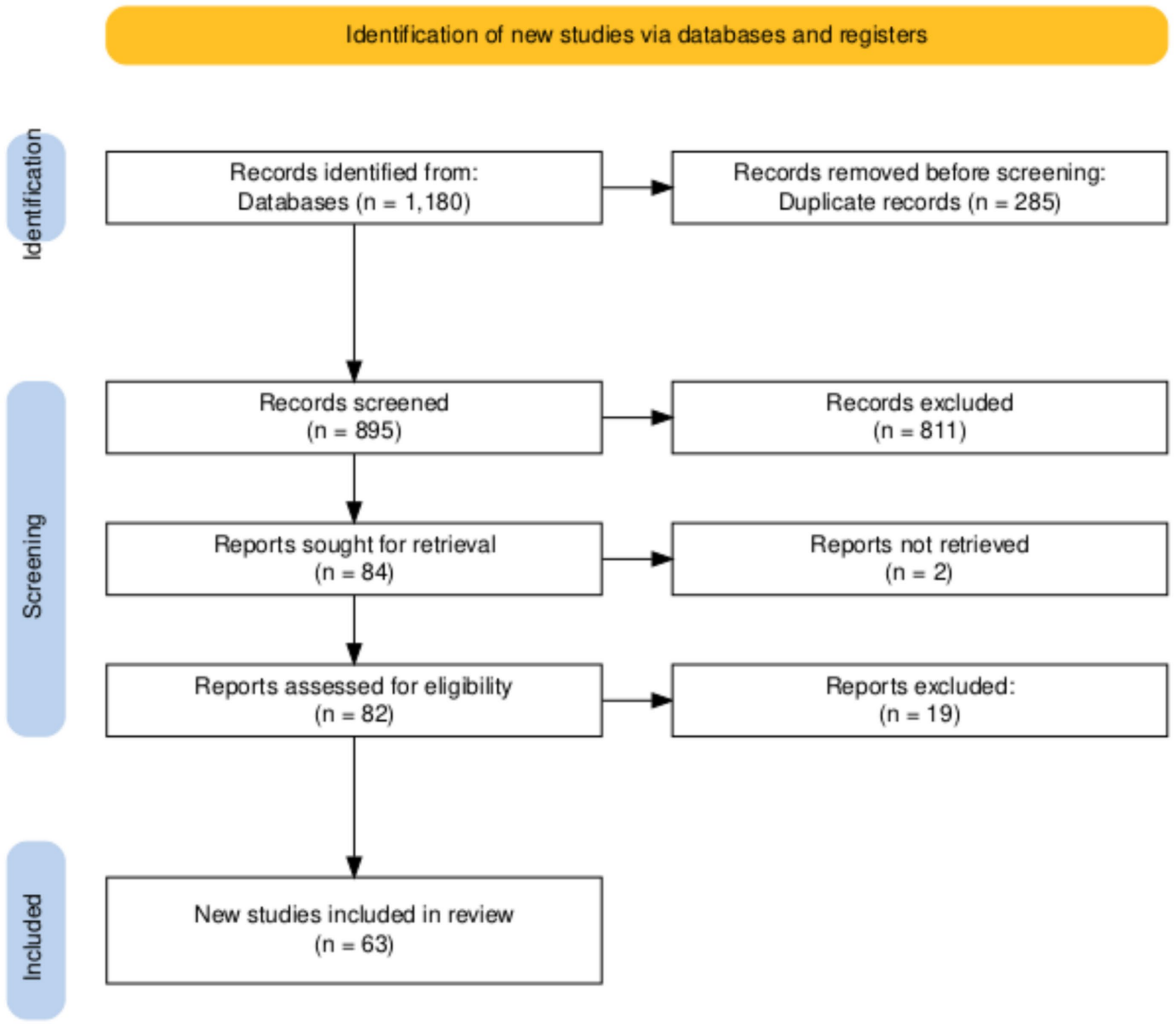
PRISMA 2020 flow diagram for study selection.

The process of study selection is illustrated in Figure 1. All records identified through the database and manual searches were compiled, and duplicates were removed. The titles and abstracts of the remaining records were screened for relevance by the authors. The full texts of potentially relevant articles were then retrieved and assessed for final eligibility against the criteria outlined above. The synthesis of the selected literature was performed narratively to construct a clinically useful framework for diagnosis and management.

## Epidemiology and demographics

The anti-GQ1b antibody syndromes are rare disorders, a fact that both complicates diagnosis and limits the feasibility of large-scale clinical trials (13). The most robust epidemiological data exist for Guillain-Barré syndrome (GBS), the most common entity within the spectrum. GBS has a well-established annual incidence of approximately 1.1–1.8 cases per 100,000 people worldwide (14–16). Its incidence increases with age, particularly in individuals over 50, and there is a slight male predominance (14, 17, 18).

The core variants of the anti-GQ1b antibody syndrome are significantly less common. Miller Fisher syndrome (MFS) is estimated to account for only about 3% of GBS cases in Western countries, although this proportion may be higher (up to 34%) in certain regions like East Asia (19). Bickerstaff Brainstem Encephalitis (BBE) is rarer still. While comprehensive global incidence data for BBE are lacking, a nationwide survey in Japan estimated its annual incidence to be approximately 0.08 per 100,000 population (5), highlighting its extreme rarity compared to GBS.

These syndromes predominantly affect adults, with some case series reporting a mean age of onset in the mid-50s (20, 21). However, they can occur at any age, including in pediatric populations, where the median age of onset is often in late childhood (22). A slight male predominance has been noted across the spectrum (17). A crucial epidemiological feature is the presence of an antecedent infection, which is reported in up to 75% of GBS cases and is also a common feature of MFS and BBE (23). The most frequently implicated pathogens include *Campylobacter jejuni*, *Haemophilus influenzae*, and—less commonly—*Mycoplasma pneumoniae* (24–26). The scarcity of these conditions underscores the importance of clinical recognition and a structured diagnostic approach for physicians who may encounter them infrequently.

## Pathophysiology: a spectrum of antibody-mediated neural injury

The diverse clinical syndromes that constitute the anti-GQ1b antibody spectrum are unified by a shared immunopathogenesis: a post-infectious, antibody-mediated assault on the nervous system (27). A comprehensive understanding of this process requires a dual focus: first, on the well-established cascade of molecular mimicry in seropositive disease, and second, on the competing hypotheses that seek to explain the significant minority of seronegative cases.

### The infectious trigger and molecular mimicry

The onset of the syndrome typically follows an antecedent upper respiratory or gastrointestinal infection by several days or weeks. The most frequently implicated pathogens are *Campylobacter jejuni*, *Haemophilus influenzae* (24–26). The lipo-oligosaccharides (LOS) on the surface of these microbes share a striking structural resemblance to gangliosides, which are abundant in human neural tissue (27–29). In its effort to clear the infection, the host’s immune system generates IgG antibodies against these microbial LOS epitopes which, due to molecular mimicry, mistakenly recognize and bind to self-gangliosides (8).

The discovery of anti-GQ1b IgG antibodies in approximately two-thirds of patients with BBE provided the crucial link to MFS and the conceptual foundation for the syndrome. The specific clinical triad of ophthalmoplegia, ataxia, and altered consciousness is a direct consequence of the high density of the GQ1b ganglioside in specific neuroanatomical locations (9, 30):

1) Oculomotor nerves (CN III, IV, VI): Leading to ophthalmoplegia.
2) Muscle spindle afferents: Disrupting proprioceptive feedback and causing ataxia.
3) Brainstem reticular activating system: Impairing arousal and causing altered consciousness.

**Figure 2:**
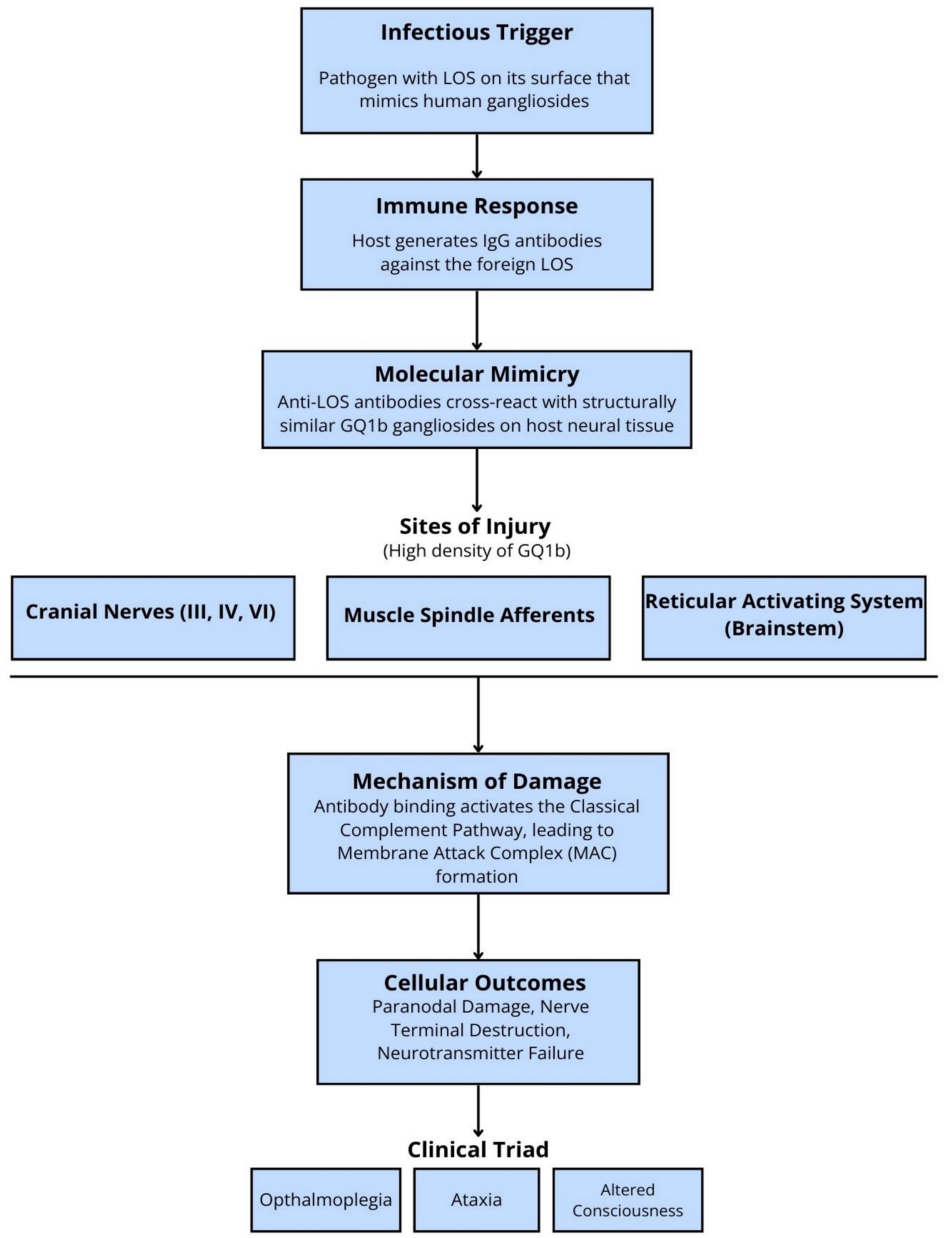
The proposed pathophysiological cascade in seropositive Bickerstaff brainstem encephalitis (BBE). BBE: Bickerstaff brainstem encephalitis; CNS: central nervous system; GSCs: ganglioside complexes; IgG: immunoglobulin G; LOS: lipo-oligosaccharides; MAC: membrane attack complex; PNS: peripheral nervous system. This flowchart illustrates the proposed mechanism of post-infectious molecular mimicry that leads to the clinical manifestations of BBE. An immune response to an antecedent infection (e.g., *Campylobacter jejuni*) generates Immunoglobulin G (IgG) antibodies against microbial lipo-oligosaccharides (LOS). Due to structural similarities, these antibodies cross-react with GQ1b gangliosides, which are highly expressed in the oculomotor nerves (cranial nerves III, IV, VI), muscle spindles, and the brainstem’s reticular activating system. This binding triggers the classical complement pathway, leading to the formation of the membrane attack complex (MAC), which causes paranodal nerve terminal injury. This targeted damage results in the classic clinical triad of ophthalmoplegia, ataxia, and altered consciousness. CNS: Central Nervous System; GSCs: Ganglioside Complexes; IgG: Immunoglobulin G; LOS: Lipo-oli gosaccharides; MAC: Membrane Attack Complex; PNS: Peripheral Nervous System.

Upon binding to these targets, anti-GQ1b IgG antibodies activate the classical complement pathway, culminating in the formation of the membrane attack complex (MAC). The MAC inserts into nerve terminal membranes, causing paranodal damage and neurotransmitter failure. This complement-mediated destruction is the final common pathway of injury in seropositive disease (31–33) (Figure 2).

### The seronegative enigma: weighing evidence for alternative pathologies

While the anti-GQ1b model is compelling, it is incomplete. As previously noted, up to one-third of patients with a classic BBE phenotype are seronegative (4, 10–12), presenting a significant conceptual challenge. This suggests that “seronegative BBE” is likely a heterogeneous condition. The evidence points to at least three non-mutually exclusive hypotheses.

First, a T-Cell-Mediated Pathology: A subset of seronegative BBE may be driven primarily by a cell-mediated process. This is supported by the higher frequency of CSF pleocytosis and T2/FLAIR hyperintense lesions on brain MRI in seronegative patients, findings more consistent with a CNS-intrinsic inflammatory process involving T-cell infiltration (4, 11, 12).

Second, Alternative or Undetected Antibodies: Seronegative patients may have a humoral-mediated disease driven by antibodies against different targets or those missed by current assays. Emerging evidence points to antibodies against ganglioside complexes (GSCs)—such as GD1a/GD1b or GD1b/GT1b—which may be pathogenic in some anti-GQ1b-negative patients (34–36).

Third, Methodological Limitations: Some cases of seronegativity may be factitious, resulting from transient antibody production, low antibody titers, or compartmentalization of antibodies within the intrathecal space (37).

### Therapeutic implications and future directions

The central role of antibodies and complement provides a direct rationale for the primary immunotherapies used in BBE: intravenous immunoglobulin (IVIg), which is thought to neutralize antibodies and inhibit complement, and plasma exchange (PE), which physically removes them (32, 38). Several critical questions remain, including definitively characterizing the T-cell repertoire in seronegative patients and elucidating the host-specific factors that dictate clinical phenotype (9, 11, 38). Answering these questions is essential for developing more targeted therapies, such as complement inhibitors, that can more precisely disrupt the final pathway of neural injury.

Ultimately, whether driven by anti-GQ1b antibodies or other mechanisms, this autoimmune assault produces a diverse array of clinical signs. The specific phenotype that emerges in a given patient is determined by the precise anatomical distribution and severity of the injury along the neuroaxis, as will be explored in the following section (39).

## Clinical manifestations: a unified spectrum of neuroaxis injury

**Figure 3:**
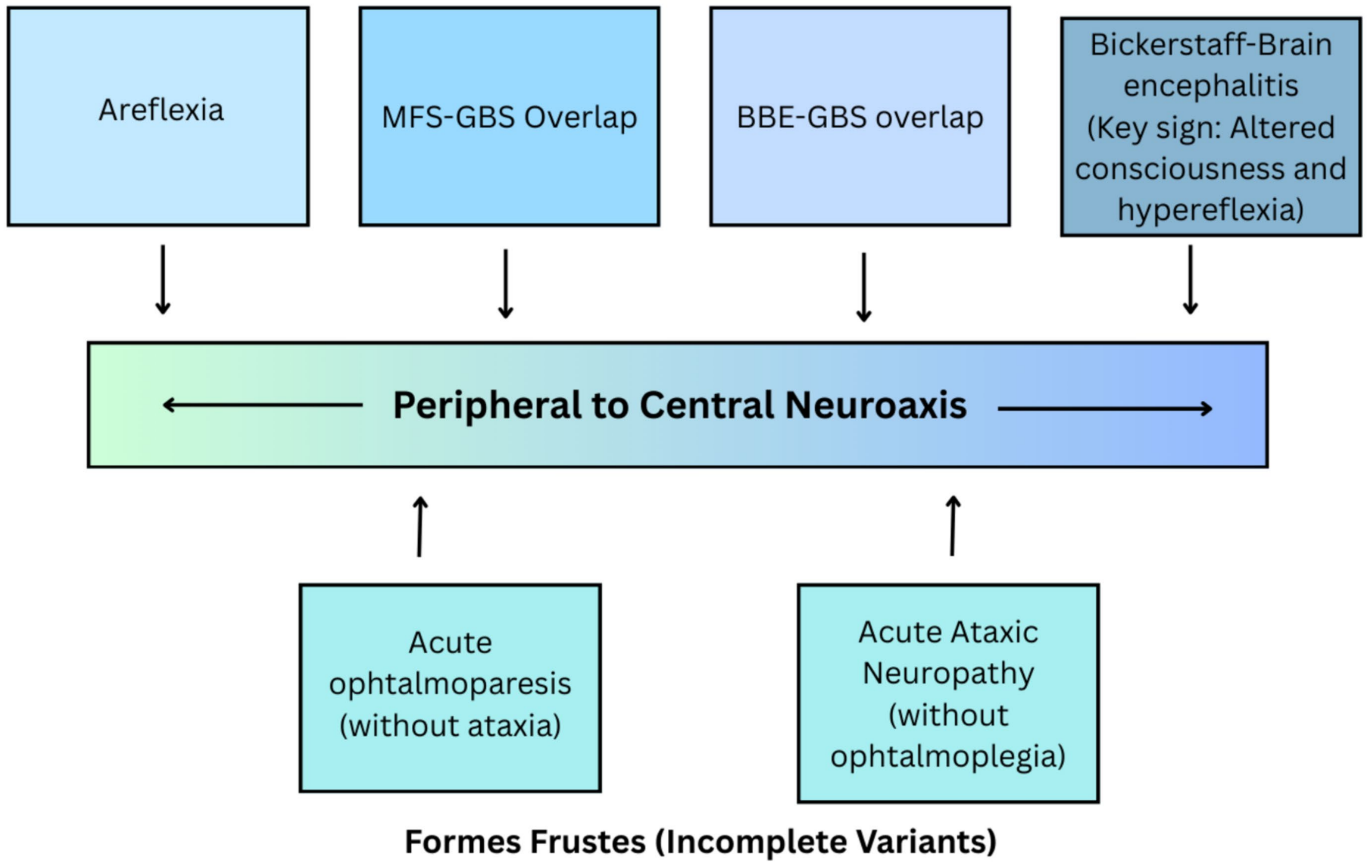
The proposed clinico-pathophysiological spectrum of the anti-GQ1b antibody syndrome. This model conceptualizes the anti-GQ1b antibody-associated disorders as a single, continuous spectrum of autoimmune injury along a peripheral-to-central neuroaxis. The spectrum is anchored by classic Miller Fisher syndrome (MFS) at the peripheral pole, characterized by ophthalmoplegia, ataxia, and areflexia, and classic Bickerstaff brainstem encephalitis (BBE) at the central pole, defined by ophthalmoplegia, ataxia, and altered consciousness with upper motor neuron signs (e.g., hyperreflexia). Between these poles lie numerous overlap syndromes, such as the BBE-Guillain-Barré syndrome (BBE-GBS) overlap, and incomplete variants (*formes frustes*), like isolated acute ophthalmoparesis. BBE: Bickerstaff Brainstem Encephalitis; GBS: Guillain-Barré Syndrome; MFS: Miller Fisher Syndrome.

The autoimmune assault on the nervous system produces a diverse array of clinical signs, best understood as variable expressions along a continuous spectrum of neuroaxis injury. The nosological ambiguity surrounding Bickerstaff Brainstem Encephalitis (BBE), Miller Fisher syndrome (MFS), and their variants has long posed a diagnostic challenge. These historically distinct syndromes are best understood not as separate entities, but as variable expressions of a single, continuous clinicopathological spectrum (40). This paradigm, unified by the anti-GQ1b antibody, provides a robust framework for interpreting clinical presentations, which are determined by the anatomical locus and extent of autoimmune injury along the peripheral-to-central neuroaxis, as illustrated in Figure 3.

### The central nervous system pole: classic Bickerstaff brainstem encephalitis

The quintessential manifestation of central nervous system (CNS) involvement is classic BBE, which anchors the central pole of the spectrum (4). Its defining clinical signature is an alteration of consciousness, ranging from mild somnolence to profound coma, reflecting dysfunction of the brainstem’s ascending reticular activating system. This is typically accompanied by bilateral external ophthalmoplegia and prominent cerebellar ataxia. The diagnosis is further solidified by the frequent presence of long-tract signs, such as hyperreflexia and extensor plantar responses, which indicate corticospinal tract involvement and distinguish BBE from purely peripheral neuropathies (10, 41).

### The peripheral nervous system pole: classic Miller Fisher syndrome

At the antithetical pole of the spectrum lies MFS, a purely peripheral syndrome defined by the classic triad of ophthalmoplegia, ataxia, and, critically, areflexia or hyporeflexia. In pure MFS, consciousness is entirely spared, and there are no signs of CNS pathology. The areflexia is the cardinal sign of peripheral nerve compromise, resulting from antibody-mediated damage to large-fiber proprioceptive afferents from muscle spindles, which disrupts the spinal reflex arc (42–44).

### The clinical continuum: overlap syndromes

The most compelling evidence for a unified spectrum comes from the significant number of patients who present with overlapping phenotypes. The most common and clinically important of these is the BBE-Guillain-Barré Syndrome (GBS) overlap, which may occur in up to 60% of patients diagnosed with BBE (10). These individuals exhibit the characteristic brainstem dysfunction of BBE, including altered sensorium, alongside the hallmark of GBS: progressive, symmetric, and areflexic flaccid quadriparesis. This mixed presentation underscores a widespread autoimmune attack affecting both central and peripheral nerve structures simultaneously (4, 8, 45).

To systematize the diagnostic approach and bring nosological clarity, Table 1 provides a comparative framework summarizing the key distinguishing features of these core phenotypes.

**Table 1:** Clinical and paraclinical differentiation of the anti-GQ1b antibody spectrum phenotypes.

Feature	Miller Fisher Syndrome (MFS)	Bickerstaff Brainstem Encephalitis (BBE)	BBE-GBS Overlap Syndrome
Consciousness	Normal/Alert	Impaired (Somnolence to Coma)	Impaired
Limb Reflexes	Areflexia/Hyporeflexia	Hyperreflexia/Normal	Areflexia/Hyporeflexia
Limb Weakness	Absent or Minimal	Absent or Minimal	Prominent, Ascending, Symmetrical
Plantar Response	Flexor	Extensor (Babinski)	Flexor (Reflecting Dominant PNS Injury)
CSF Pleocytosis	Uncommon (~5%)	Common (~32%)	Variable
Brain MRI	Typically Normal	Abnormalities More Frequent (e.g., brainstem hyperintensities)	Abnormalities as in BBE May Be Present

BBE, Bickerstaff Brainstem Encephalitis; CSF, Cerebrospinal Fluid; GBS, Guillain-Barré Syndrome; MFS, Miller Fisher Syndrome; PNS, Peripheral Nervous System.

### Incomplete phenotypes and focal variants

The spectrum is further populated by incomplete expressions (*formes frustes*) of the core syndromes, which are part of the same pathogenic family. These focal variants, often associated with anti-GQ1b antibodies, include isolated acute ophthalmoparesis (without ataxia or areflexia) and acute ataxic neuropathy. Recognizing these limited presentations is crucial, as they broaden the differential diagnosis and highlight the role of serological testing when the full clinical triad is absent (4, 46).

Accurately placing a patient on this clinical spectrum, from a focal variant to a widespread overlap syndrome, requires a systematic diagnostic approach that integrates clinical acumen with the strategic use of multimodal investigations.

## Diagnostic approach: integrating clinical acumen with multimodal investigations

**Figure 4:**
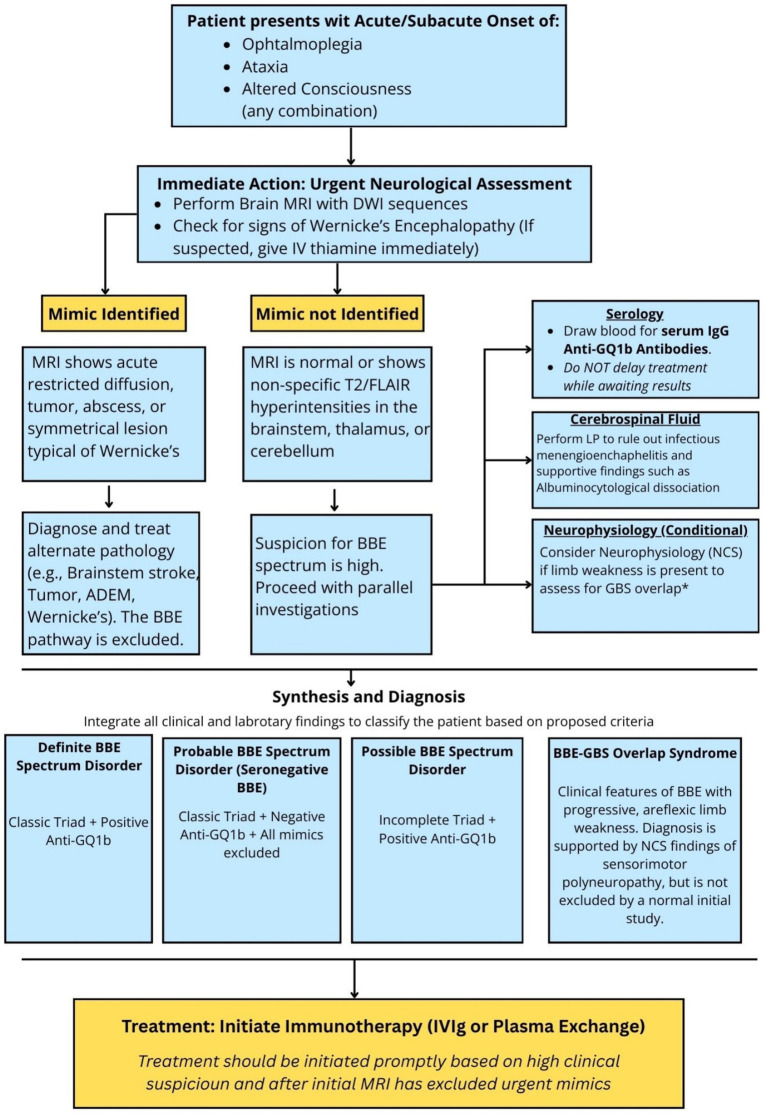
Proposed diagnostic algorithm for suspected Bickerstaff brainstem encephalitis (BBE). This flowchart outlines a systematic, step-wise approach for the clinician evaluating a patient with an acute onset of ophthalmoplegia, ataxia, and altered consciousness. The algorithm prioritizes the urgent exclusion of time-sensitive mimics (e.g., brainstem stroke) via brain magnetic resonance imaging (MRI) and confirms the diagnosis through a combination of clinical findings and serology. A key component of the evaluation is the assessment for overlap with Guillain-Barré syndrome (GBS) in patients with limb weakness. *While nerve conduction studies (NCS) are essential for this purpose, it is crucial to recognize that electrophysiological abnormalities may take days or weeks to develop. Therefore, a normal finding on an initial NCS does not exclude an evolving BBE-GBS overlap syndrome, and repeat testing should be considered if clinical suspicion persists. **BBE: Bickerstaff Brainstem Encephalitis; CNS: Central Nervous System; CSF: Cerebrospinal Fluid; GQ1B: Ganglioside antigen; LP: Lumbar Puncture; MFS: Miller Fisher Syndrome; MRI: Magnetic Resonance Imaging; NCS: Nerve Conduction Studies.

The diagnosis of Bickerstaff Brainstem Encephalitis (BBE) and its related syndromes is a synthetic exercise in clinical reasoning, resting on astute recognition supported by a strategic application of multimodal investigations (38, 47). We propose a structured diagnostic algorithm, outlined in Figure 4, to guide this process. The algorithm emphasizes the dual goals of confirming the BBE spectrum phenotype while aggressively excluding critical, time-sensitive mimics.

### The primacy of clinical recognition

The diagnostic pathway is initiated by a high index of suspicion based on the acute or subacute onset of the classic triad: ophthalmoplegia, ataxia, and altered sensorium (48). The presence of upper motor neuron signs, such as hyperreflexia or extensor plantar responses, strongly points toward the CNS involvement characteristic of BBE (4). Conversely, the finding of areflexia should prompt immediate consideration of an overlap syndrome with GBS (49).

### Serological testing: the pathophysiological anchor

The detection of serum IgG anti-GQ1b antibodies is the most specific diagnostic test, providing a direct link to the underlying autoimmune pathophysiology. A positive anti-GQ1b antibody test in a patient with a compatible clinical syndrome is strongly confirmatory. However, their absence does not exclude the diagnosis. Therefore, “seronegative BBE” is a valid and necessary clinical diagnosis that relies on the rigorous exclusion of other causes. Crucially, immunotherapy should not be withheld or delayed while awaiting serology results if clinical suspicion is high (32).

### Neuroimaging: the indispensable role in exclusion

Magnetic resonance imaging (MRI) of the brain is a mandatory investigation for every suspected case of BBE; its primary goal is not to confirm the diagnosis but to definitively rule out acute brainstem pathologies that constitute neurological emergencies, such as basilar artery thrombosis, tumors, or abscesses. In most BBE patients, MRI is normal, and this is fully compatible with the diagnosis. When abnormalities are detected—reported in roughly one-third or fewer of cases—they usually appear as non-specific, often transient T2-weighted or FLAIR hyperintensities involving the brainstem, and in some series also the thalamus or cerebellum. Abnormal MRI findings seem to be more common in anti-GQ1b–negative or atypical BBE, but the underlying immunopathology of this subgroup (for example, a more T-cell–mediated process) remains speculative (50).

### Ancillary investigations: supportive evidence

Cerebrospinal fluid (CSF) analysis and electrophysiology provide valuable supportive data, primarily by excluding other etiologies and characterizing overlap syndromes. The classic finding of albuminocytologic dissociation is supportive but is only present in a minority of BBE cases (around 25%). The main role of CSF analysis is to exclude infectious meningoencephalitis. Nerve conduction studies (NCS) are often normal in pure BBE but are essential in patients with limb weakness to identify a concurrent sensorimotor polyneuropathy, thereby confirming a BBE-GBS overlap syndrome. Crucially, it must be emphasized that electrophysiological abnormalities may take days or even weeks to develop. Therefore, normal findings on an initial nerve conduction study do not exclude a BBE-GBS overlap syndrome, and repeat testing may be necessary if clinical suspicion for peripheral nerve involvement persists. An electroencephalogram (EEG) typically shows non-specific diffuse slowing and is mainly useful to exclude non-convulsive status epilepticus as the cause for altered consciousness (4, 45).

### From differential to definition: proposed clinical criteria for the BBE spectrum

To bring nosological clarity and standardize diagnosis for both clinical and research purposes, we propose a structured framework based on our spectrum model. The criteria presented in Table 2 are designed to encompass the full range of phenotypes, including seronegative disease and incomplete variants, and help synthesize the clinical and investigational findings into a definitive classification.

**Table 2:** Proposed clinical criteria for Bickerstaff Brainstem Encephalitis (BBE) spectrum disorders.

Category	Required clinical features	Required laboratory and imaging features
Definite BBE Spectrum Disorder	Acute/subacute onset of the classic triad: Bilateral external ophthalmoplegia Ataxia Altered consciousness (somnolence to coma)	AND Positive serum IgG anti-GQ1b antibodies Exclusion of mimics via appropriate investigations (esp. brain MRI)
Probable BBE Spectrum Disorder (*Seronegative BBE*)	Acute/subacute onset of the classic triad: Bilateral external ophthalmoplegia Ataxia Altered consciousness (somnolence to coma)	AND Negative serum IgG anti-GQ1b antibodies Rigorous exclusion of all critical mimics (e.g., normal DWI on MRI, negative CSF infectious studies)
Possible BBE Spectrum Disorder	Incomplete clinical presentation (e.g., only two of the classic triad, such as ataxic hypersomnolence without ophthalmoplegia)	AND Positive serum IgG anti-GQ1b antibodies Exclusion of mimics via appropriate investigations

BBE, Bickerstaff Brainstem Encephalitis; CSF, Cerebrospinal Fluid; DWI, Diffusion-Weighted Imaging; GQ1B, Ganglioside antigen; IgG, Immunoglobulin G; MRI, Magnetic Resonance Imaging.

Having established a diagnosis and classified the patient within the anti-GQ1b spectrum, the clinician’s next critical task is to initiate appropriate therapy to mitigate neurological injury and support recovery.

## Therapeutic strategy and future horizons in the anti-GQ1b spectrum

**Figure 5:**
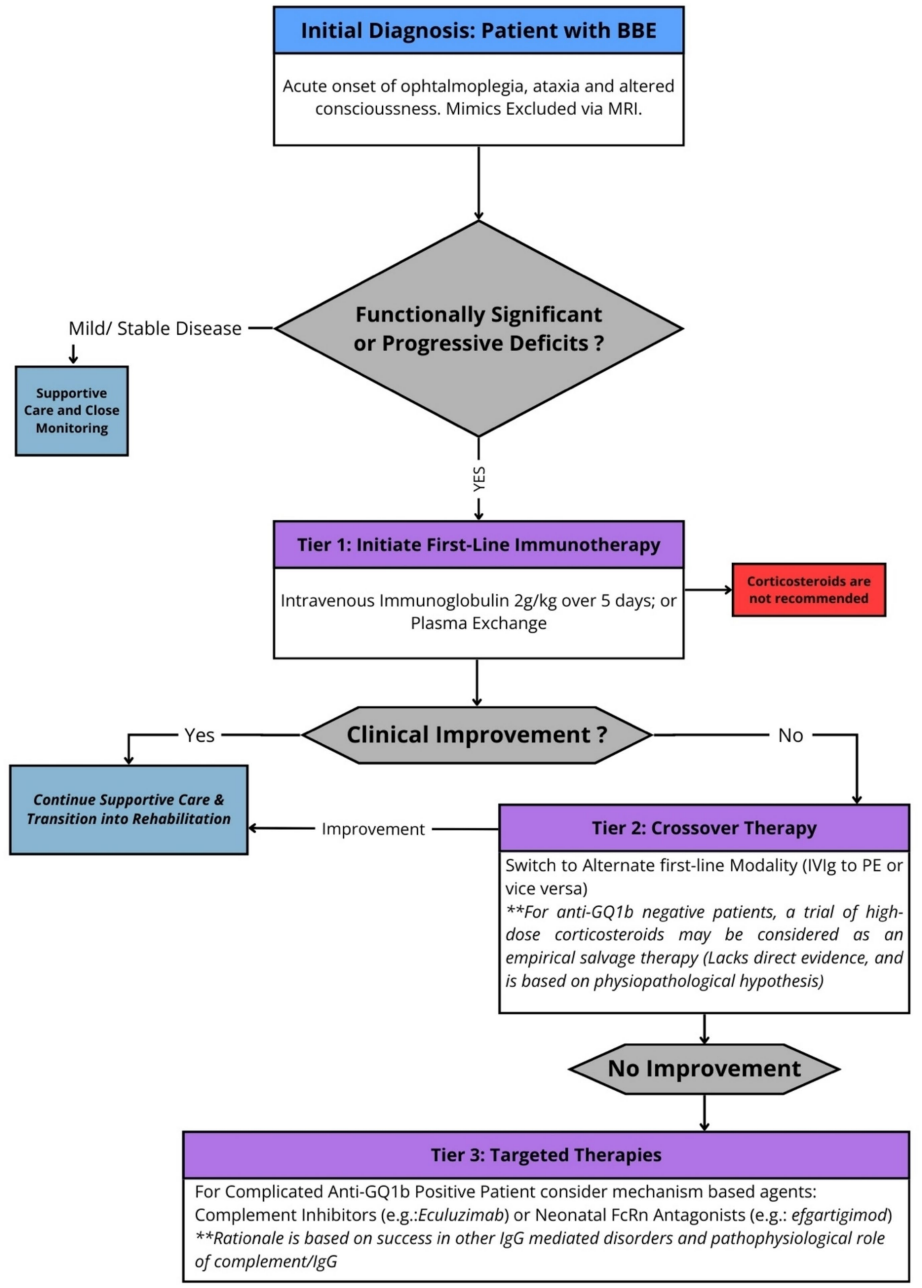
A proposed risk-stratified treatment algorithm for Bickerstaff brainstem encephalitis (BBE). The algorithm begins with the diagnosis of BBE in a patient with functionally significant deficits. Tier 1 involves first-line immunotherapy with either intravenous immunoglobulin (IVIg) or plasma exchange (PE), based on extrapolated evidence from Guillain-Barré syndrome trials. Corticosteroid monotherapy is not recommended. If the patient fails to improve (refractory disease), Tier 2 involves a crossover to the alternate first-line therapy. For patients who remain refractory after both IVIg and PE, further management is guided by anti-GQ1b serostatus and pathophysiological reasoning. For anti-GQ1b negative patients, a trial of high-dose corticosteroids may be considered as an empirical salvage therapy. For severe, anti-GQ1b positive patients, emerging targeted therapies such as complement inhibitors or neonatal Fc receptor (FcRn) antagonists represent a rational, albeit investigational, next step (Tier 3). BBE: Bickerstaff brainstem encephalitis; IVIg: intravenous immunoglobulin; PE: plasma exchange; RCTs: randomized controlled trials.

The management of Bickerstaff Brainstem Encephalitis (BBE) remains one of the great evidence-free zones in clinical neuroimmunology. In the absence of prospective, randomized controlled trial data for BBE itself (11), clinicians are forced to navigate therapeutic decisions by extrapolating from the related Guillain-Barré syndrome (GBS) literature—a reasonable but imperfect surrogate (51, 52). Optimal management is therefore not a matter of following established guidelines, but of applying a risk-stratified paradigm based on a nuanced understanding of the underlying pathophysiology and the patient’s clinical trajectory. To provide a clear clinical framework, we propose a tiered approach to immunotherapy, moving from established practice to more targeted strategies in severe or refractory cases, a process schematized in Figure 5.

The first tier of therapy applies to any patient with functionally significant or progressive deficits, such as an inability to ambulate or a declining level of consciousness, for whom immediate intervention is mandatory. Based on extensive GBS data, Intravenous Immunoglobulin (IVIg), administered at a dose of 2 g/kg over 5 days, and Plasma Exchange (PE), typically involving five sessions, are the two equally effective first-line modalities (53, 54). IVIg is often favored for its logistical simplicity and its known mechanisms of complement inhibition and antibody neutralization (55). PE, which works by directly removing pathogenic autoantibodies, is an equally valid alternative and is often preferred in patients with contraindications to large volume infusions, such as severe heart failure (56). Importantly, high-quality evidence from multiple RCTs in GBS has demonstrated that corticosteroid monotherapy lacks efficacy and is therefore not recommended as a primary treatment in BBE and its related syndromes (57).

A subset of patients either fail to respond to first-line therapy or present with a fulminant course, constituting a neurological emergency and requiring escalation to Tier 2 management. Refractory disease is typically defined as a lack of clinical stabilization or improvement within 7–10 days of completing a first-line course. Here, evidence is anecdotal but logically supports a crossover to the alternate first-line modality, such as initiating PE following IVIg failure (32). It is in these refractory cases that the serostatus of the patient becomes a critical factor in further decision-making. For the anti-GQ1b negative patient who fails to respond to both IVIg and PE, profound uncertainty remains. Given the hypothesis that a distinct, T-cell-mediated process may be at play, a trial of high-dose intravenous corticosteroids could be justifiable as a last-resort salvage therapy. This recommendation is based solely on pathophysiological reasoning and must be considered empirical and reserved for deteriorating patients who have exhausted standard immunotherapies.

For the patient with confirmed anti-GQ1b antibodies and a severe or refractory course, Tier 3 conceptually offers emerging therapies that directly target the proposed disease mechanism. This mechanism-based approach moves beyond broad immunosuppression toward precision immunotherapy. Given the role of complement-mediated damage in seropositive BBE (58), targeted C5 complement inhibitors like eculizumab represent a highly rational option (59). Another novel class of drugs, Neonatal Fc receptor (FcRn) antagonists such as efgartigimod, accelerates the degradation of pathogenic IgG antibodies and maybe a promising area for investigation in life-threatening BBE, given its efficacy in other IgG-mediated disorders (60–63).

Finally, the therapeutic mandate extends far beyond the acute phase into long-term multidisciplinary rehabilitation. While motor recovery is often good, clinicians must address the hidden morbidity—including central fatigue, vestibular dysfunction, and cognitive deficits—that frequently persists as a sequela of brainstem inflammation. A dedicated team including neurology, physiatry, and neuropsychology is essential for optimizing long-term outcomes and quality of life. The current reliance on extrapolated evidence and the promise of targeted therapies underscores the urgent need for a dedicated research agenda to fill the evidence void.

## Strengths and limitations of this review

The primary strength of this review is its clinically focused, integrative synthesis of a complex and heterogeneous body of literature. By consolidating evidence from epidemiology, immunopathogenesis, and clinical neurology, it provides a structured framework intended to help practicing clinicians navigate the diagnostic and therapeutic challenges posed by the anti-GQ1b antibody spectrum. The proposed diagnostic and therapeutic algorithms aim to translate this synthesized evidence into a practical, actionable guide.

However, this review has important limitations, which reflect the constraints of the available evidence base for these rare disorders.

1) Methodology: As a narrative review, the selection of literature is based on expert curation rather than a pre-specified systematic protocol. This approach is susceptible to selection bias and does not have the methodological rigor of a formal systematic review or meta-analysis.
2) Limited primary evidence: The understanding of BBE is built predominantly on retrospective case series and individual reports. The complete absence of prospective, randomized controlled trials (RCTs) specific to BBE means that the field lacks high-quality evidence to definitively guide practice.
3) Extrapolation of therapeutic evidence: The therapeutic strategies outlined in this review, while representing the current standard of care, are based almost entirely on extrapolation from RCTs conducted in Guillain-Barré syndrome. The true efficacy of these immunotherapies specifically within the BBE population remains unproven.
4) The “Seronegative Enigma”: The underlying mechanisms in the substantial minority of seronegative BBE cases remain speculative. This knowledge gap complicates diagnosis—forcing it to be one of rigorous exclusion—and currently hinders the development of targeted therapies for this cohort.

Acknowledging these limitations is crucial for contextualizing the conclusions of this review and underscores the urgent need for a collaborative research agenda to move the field toward evidence-based precision.

## Conclusion: a manageable clinical challenge

Bickerstaff Brainstem Encephalitis is best understood not as an isolated curiosity, but as the central nervous system pole of a continuous anti-GQ1b antibody-mediated spectrum that includes Miller Fisher syndrome and Guillain-Barré syndrome. This review has synthesized the current clinical and pathophysiological evidence to provide a structured, integrative framework for clinicians. Adopting a spectrum-based perspective is not merely an academic exercise but a clinical necessity that clarifies diagnostic logic, rationalizes therapeutic decisions, and unifies the understanding of these heterogeneous presentations.

This synthesized view has direct implications for clinical practice. First, it reinforces the need for an integrated diagnostic strategy where a high index of clinical suspicion—even in the absence of anti-GQ1b antibodies—is the primary driver for excluding critical mimics and initiating care. Second, the compelling pathophysiological rationale extrapolated from GBS supports the prompt initiation of immunotherapy with IVIg or plasma exchange in all but the mildest cases to mitigate neurological injury. Finally, effective management must extend beyond the acute phase, incorporating a multidisciplinary rehabilitation plan to address the full spectrum of potential physical and neurocognitive sequelae.

The synthesis of current knowledge also illuminates a clear path for future investigation. A pressing research agenda must now focus on answering three key questions:

What are the primary pathogenic drivers in seronegative BBE? This requires advanced immunological approaches to identify novel antibody targets or define the T-cell repertoires responsible for CNS inflammation.

How can a direct evidence base for treatment in BBE be established? This necessitates international patient registries to enable prospective studies and adaptive platform trials comparing IVIg, plasma exchange, and targeted therapies like complement inhibitors.

What is the true long-term burden of BBE? This requires outcome studies using validated, patient-reported measures to characterize the trajectory of cognitive fatigue, executive dysfunction, and quality of life.

By pursuing this focused agenda, the clinical and scientific communities can continue to transform Bickerstaff Brainstem Encephalitis from a complex syndrome into a solvable neuroimmunological challenge.
